# LDLRAD2 overexpression predicts poor prognosis and promotes metastasis by activating Wnt/β-catenin/EMT signaling cascade in gastric cancer

**DOI:** 10.18632/aging.102359

**Published:** 2019-10-24

**Authors:** Yucai Wei, Fan Zhang, Tong Zhang, Yating Zhang, Hao Chen, Furong Wang, Yumin Li

**Affiliations:** 1 Department of Surgical Oncology, Lanzhou University Second Hospital, Lanzhou 730000, China; 2 Key Laboratory of Digestive System Tumors of Gansu Province, Lanzhou University Second Hospital, Lanzhou 730000, China; 3 Department of Hepatic Surgery, Liver Transplant Center, The Third Affiliated Hospital of Sun Yat-sen University, Guangzhou 510630, China; 4 Department of Pathology, Lanzhou University Second Hospital, Lanzhou 730000, China

**Keywords:** LDLRAD2, prognosis, metastasis, β-catenin, gastric cancer

## Abstract

The therapeutic strategies for advanced gastric cancer (GC) remain unsatisfying and limited. Therefore, it is still imperative to fully elucidate the mechanisms underlying GC aggressive progression. The prognostic value and biological functions of low density lipoprotein receptor class A domain containing protein 2 (LDLRAD2) in GC have never been studied yet. We found that LDLRAD2 expression was significantly upregulated in GC and closely correlated with poor prognosis in GC patients. Functionally, LDLRAD2 promoted epithelial-mesenchymal transition, migration and invasion, and metastasis of GC cells. Mechanistically, LDLRAD2 interacted with and inhibited Axin1 from binding to cytoplasmic β-catenin, which facilitated the nuclear translocation of β-catenin, thereby activating Wnt/β-catenin pathway. Inhibition of β-catenin activity markedly abolished LDLRAD2-induced migration, invasion and metastasis. Together, these results suggested that LDLRAD2 contributed to invasion and metastasis of GC through activating Wnt/β-catenin pathway. LDLRAD2/ Wnt/β-catenin axis may be a potential therapeutic target for GC treatment.

## INTRODUCTION

Gastric cancer (GC), a lethal disease, prevails worldwide [1]. In china, GC is the second most common cancer and the second leading cause of cancer-related deaths [2, 3]. High incidence of death resulted from GC is mainly attributed to the lack of early diagnosis [2, 3]. Usually, GC patients diagnosed at an advanced stage suffer from distant invasion and metastasis, which largely increased cancer-associated deaths [4, 5]. Chemotherapy is the most common treatment for GC patients in China, but its efficacy is limited [6, 7]. Targeted therapy, as a new strategy, has been expected to a novel promising strategy to treat cancer patients [8, 9]. For instance, human epidermal growth factor receptor 2 (HER2) and vascular endothelial growth factor receptor (VEGFR) are two key prognostic biomarkers in GC [10, 11] and their monoclonal antibodies have been widely used to treat advanced GC patients and bring much survival benefit [12]. However, not all patients could benefit from anti-HER2 or anti-VEGFR monoclonal antibody and the monoclonal antibodies are not suitable for GC patients with negative HER2 or VEGFR expression [12]. Therefore, it is imperative to further elucidate the molecular mechanisms underlying GC invasion and metastasis fully so as to identify novel effective therapeutic targets [13].

During epithelial-to-mesenchymal transition (EMT) process, epithelial cells lose their apical-basal polarity and cell-cell adhesion, ultimately transforming into the invasive mesenchymal cells [14]. EMT is accompanied by a reduced expression of epithelial markers including E-cadherin and γ-catenin, and meanwhile by an increased expression of mesenchymal markers such as N-cadherin and vimentin [14, 15]. EMT plays an important role in promoting tumor invasion and metastasis [15, 16]. Wnt/β-catenin signaling pathway has been demonstrated to regulate EMT of tumor cells [15–17]. Nuclear β-catenin accumulation is a hallmark of the activation of Wnt/β-catenin pathway [17]. Particularly, it was reported that nuclear β-catenin accumulation occurred in the invasive fronts of primary tumors, suggesting that activation of Wnt/β-catenin pathway closely correlated with tumor invasion and metastasis [18, 19]. Moreover, a body of evidence showed that activation of Wnt/β-catenin pathway could facilitate EMT to promote invasion and metastasis of various tumors [20–24]. Therefore, in order to develop novel therapeutic target for GC, it is imperative to elucidate the upstream molecular mechanisms underlying the activation of Wnt/β-catenin/EMT axis.

*LDLRAD2* (Low density lipoprotein receptor class A domain containing 2) gene, located on chromosome 1p36.12, is mainly expressed on cellular membrane. To date, the expression status, prognostic value and biological functions of LDLRAD2 in GC has never been investigated. In this study, we found that LDLRAD2 expression was highly expressed in GC tissues and cell lines, which was significantly related to unfavorable prognosis in GC patients. Additionally, it was observed that LDLRAD2 promoted epithelial-mesenchymal transition, in vitro migration and invasion, and in vivo metastasis of GC cells. Mechanistically, LDLRAD2-induced GC progression was dependent on the activation of Wnt/β-catenin pathway. Collectively, these findings revealed that LDLRAD2 facilitated migration, invasion and metastasis of GC by activating Wnt/β-catenin/EMT axis, suggesting that LDLRAD2/ Wnt/β-catenin axis may serve as a potential therapeutic target for GC treatment.

## RESULTS

### High LDLRAD2 expression correlates with poor prognosis and unfavorable clinical features in GC patients

**Figure 1:**
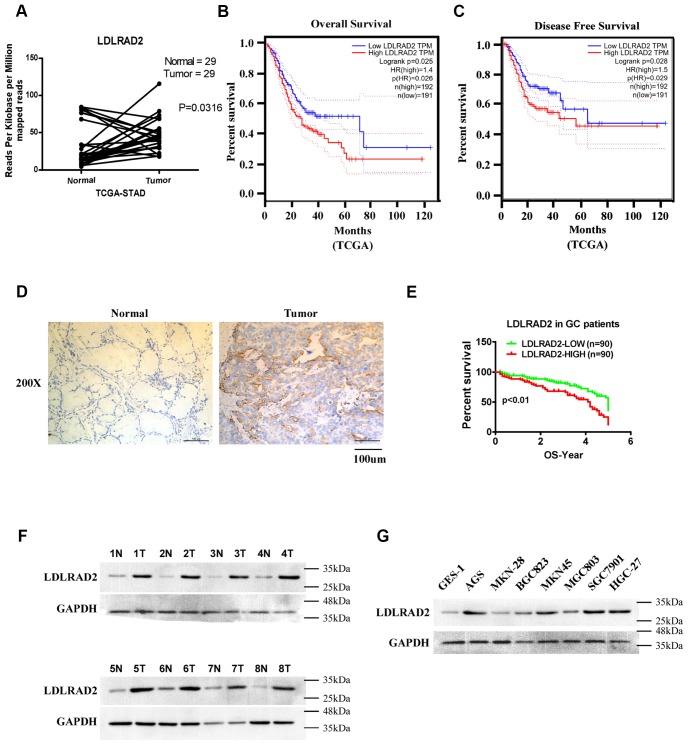
**High LDLRAD2 expression correlates with poor prognosis and unfavorable clinical features in GC patients.** Bioinformatics analysis showed that LDLRAD2 mRNA expression was significantly higher in GC samples compared with normal samples (**A**). High LDLRAD2 expression was closely correlated with short overall survival (**B**) and disease-free survival (**C**). Immunohistochemical staining showed that LDLRAD2 expression was higher in GC samples than normal control (**D**). Kaplan-Meier survival analysis of 180 patients from our clinical center suggested that patients with high LDLRAD2 expression had a decreased overall survival (**E**). Western blotting analysis further confirmed that LDLRAD2 expression is upregulated in human GC (**F**) and GC cell lines (**G**). Three independent experiments were performed. *p < 0.05, **p < 0.01, ***p < 0.001.

To explore the expression status and prognostic value of LDLRAD2 in GC, dataset downloaded from The Cancer Genome Atlas (TCGA). We found that mRNA expression of LDLRAD2 was significantly upregulated in GC samples compared with normal samples (Figure 1A) and it was inversely correlated with overall survival (Figure 1B) and disease-free survival (Figure 1C) of GC patients. To validate the results of our bioinformatics analysis, we detected LDLRAD2 expression level in 180 GC sample tissues from our clinical center using immunohistochemical staining method. Consistently, the results of immunohistochemical staining also showed that LDLRAD2 expression was highly expressed in tumor tissues (Figure 1D). To assess the prognostic significance of LDLRAD2, we divided 180 cases into high and low groups according to the median value of immunohistochemical staining scores for LDLRAD2 expressions. We found that patients with high LDLRAD2 expression had shorter overall survival (Figure 1E) than those with low LDLRAD2 expression, which was also in line with the results of bioinformatics analysis. Additionally, our western blotting analysis also showed that LDLRAD2 expression was significantly upregulated in GC tissues (Figure 1F) and GC cell lines compared with their counterparts (Figure 1G). Furthermore, we analyzed the correlation between LDLRAD2 expression and clinical features of 180 GC cases. We observed that high LDLRAD2 expression was significantly associated with aggressive features such as advanced TNM stage, positive lymph node metastasis and distant metastasis (Table 1). Together, our results demonstrated that LDLRAD2 was highly expressed in GC, which may predict poor prognosis in GC patients.

**Table 1:** The relationship between LDLRAD2 expression and clinicopathological features of patients with gastric cancer.

	**LDLRAD2 expression**		
**Clinicopathological features**	**Low**	**High**	**P value (χ2 test)**
**Age (y)**			0.520
≤50	30	26	
>50	60	64	
**Gender**			0.739
Male	64	66	
Female	26	24	
**Lauren**			0.230
Intestinal	12	18	
Diffuse	78	72	
**Clinical stage**			**0.013**
I	8	2	
II	27	16	
III	36	38	
IV	19	34	
**T classification**			0.151
T1+T2+T3	24	16	
T4	66	74	
**N classification**			**<0.001**
N0	33	13	
N1-N3	57	77	
**Distant metastasis**			**0.023**
M0	84	74	
M1	6	16	

### LDLRAD2 promotes migration and invasion in vitro of GC cells

**Figure 2:**
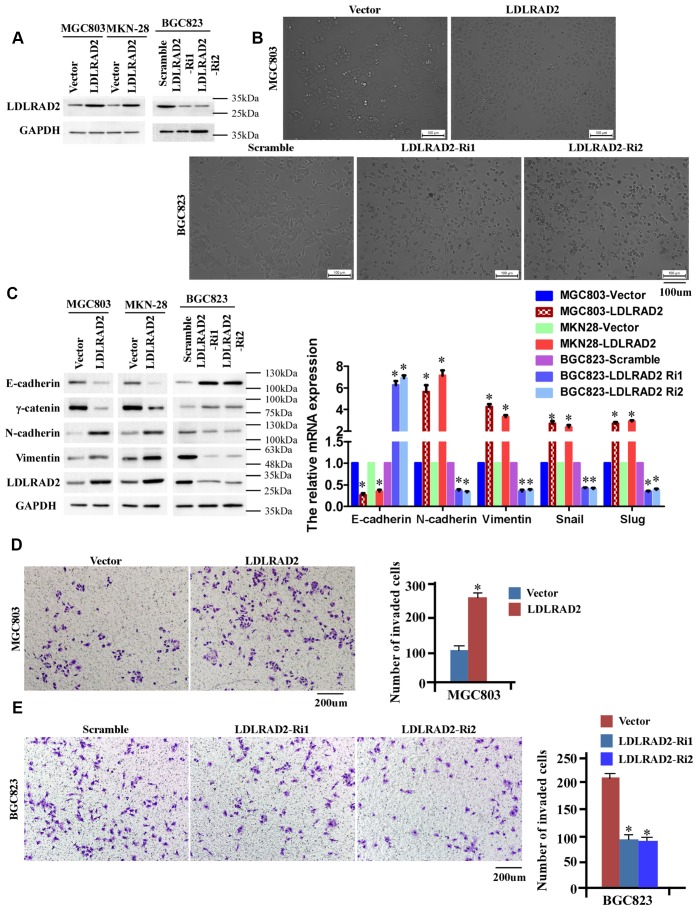
**LDLRAD2 promotes in vitro migration and invasion of GC cells.** Overexpression of LDLRAD2 in MGC-803 and MKN28 cell lines and silenced LDLRAD2 in BGC823 cell line (**A**). Overexpression of LDLRAD2 significantly enhanced morphological characteristics of EMT of MGC-803 cell line, while silence of LDLRAD2 significantly inhibited morphological characteristics of EMT of BGC823 cell line (**B**). Western blotting analysis showed that the expression levels of epithelial cell markers such as E-cadherin and γ-catenin were dramatically decreased, while mesenchymal cell markers such as N-cadherin and vimentin were significantly increased in LDLRAD2-overexpression cell lines (**C**). The expression levels of epithelial cell markers were increased, while mesenchymal cell markers were decreased in LDLRAD2-silenced cell line, (**C**). LDLRAD2-overexpression MGC-803 cell exhibited significantly enhanced invasive capability (**D**), while LDLRAD2-silenced BGC-823 cell had dramatically decreased invasive capability (**E**).

As the results above shown, MGC-803 and MKN-28 cell lines have the lowest LDLRAD2 expression, while BGC-823 cell line has a relatively high LDLRAD2 expression (Figure 1G). Hence, to investigate the migration and invasion of LDLRAD2 in GC, we stably overexpressed LDLRAD2 in MGC-803 and MKN28 cell lines and silenced LDLRAD2 in BGC823 cell line (Figure 2A). Firstly, we determined whether LDLRAD2 expression has an effect on EMT, considering the role of epithelial-mesenchymal transition (EMT) in tumor invasion. GSEA-GO analysis indicated that LDLRAD2 expression was closely related to EMT of GC cells (Supplementary Figure 1). Consistently, our experiments in vitro also showed that overexpression of LDLRAD2 significantly enhanced morphological characteristics of EMT of MGC-803 cell line (Figure 2B), which was featured with a scattered distribution of cells in the culture and a spindle- or star-like morphology of the cells. Instead, silence of LDLRAD2 inhibited morphological characteristics of EMT of BGC823 cell line (Figure 2B). To further support the role of LDLRAD2 in regulating EMT of GC cells, we used western blot to detect the expression levels of EMT-associated makers after overexpression or silence of LDLRAD2. In line with morphological alterations, our western blotting analysis showed that the expression levels of epithelial cell markers such as E-cadherin and γ-catenin were dramatically decreased, while mesenchymal cell markers such as N-cadherin and vimentin were significantly increased in LDLRAD2-overexpression cell lines (Figure 2C). Inversely, in LDLRAD2-silence cell line, the expression levels of epithelial cell markers and mesenchymal cell markers were increased and decreased, respectively (Figure 2C). Additionally, we also observed the similar effects of LDLRAD2-overexpression or LDLRAD2-silence on the mRNA levels of the EMT markers, including E-cadherin, N-cadherin, vimentin, Snail and Slug (Figure 2C). Subsequently, we performed Transwell assay to detect the in vitro migratory and invasive capabilities of GC cell lines. Consistent with results regarding EMT, it was observed that LDLRAD2-overexpression MGC-803 cell exhibited significantly enhanced invasive capability (Figure 2D), while LDLRAD2-silence BGC-823 cell exhibited dramatically decreased invasive capability (Figure 2E). Besides, we also observed the similar results of migration and invasion when LDLRAD2 was overexpressed or silenced in MKN-28 cells (Supplementary Figure 2). Taken together, these results demonstrated that LDLRAD2 overexpression promotes in vitro migration and invasion of GC cells probably by enhancing EMT.

### LDLRAD2 promotes GC metastasis in vivo

**Figure 3:**
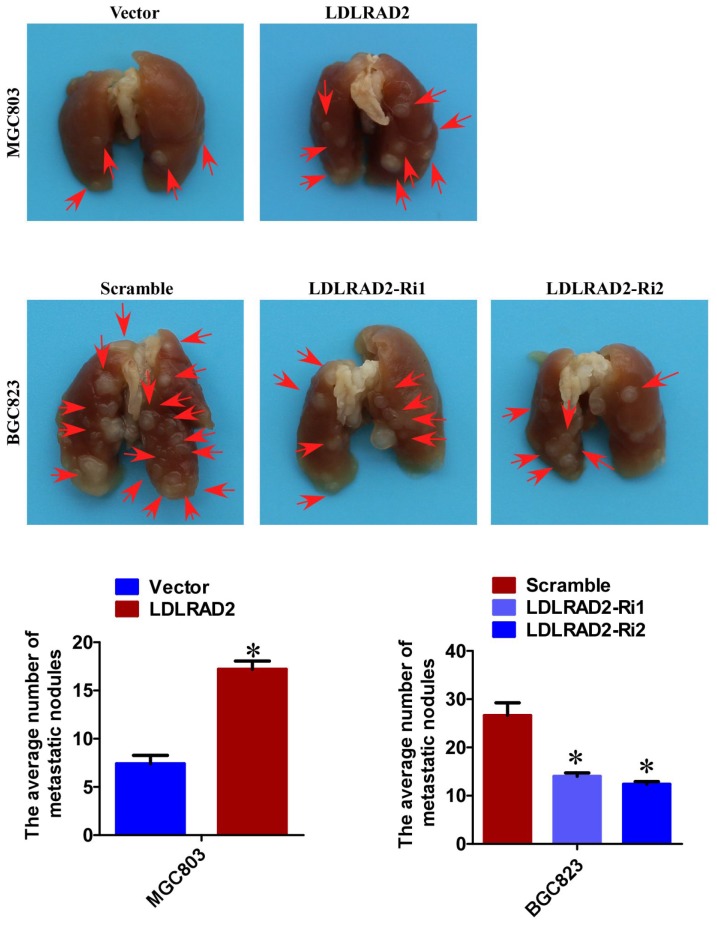
**LDLRAD2 promotes GC metastasis in vivo.** The number of lung metastatic nodules from mice injected with LDLRAD2-overexpresion MGC-803 cell was much more than that from control mice, while the number of lung metastatic nodules from mice injected with LDLRAD2-silenced BGC-823 cell was less than that from control mice.

Based on the role of LDLRAD2 in facilitating migration and invasion, we further explored whether LDLRAD2 could promote GC metastasis in vivo. We injected LDLRAD2-overexpresion MGC-803 cell and LDLRAD2-silence BGC-823 cell, as well as their corresponding vector/scramble control cells into nude mice by tail vein, separately, to explore the role of LDLRAD2 in GC metastasis in vivo. Four weeks later, the mice were sacrificed and the lung metastatic nodules were counted. Remarkably, the number of lung metastatic nodules from mice injected with LDLRAD2-overexpresion MGC-803 cell was more than that from control mice, while the number of lung metastatic nodules from mice injected with LDLRAD2-silenced BGC-823 cell was less than that from control mice, suggesting that LDLRAD2 could promote GC metastasis in vivo (Figure 3).

### Activation of Wnt/β-catenin signaling pathway is involved in LDLRAD2-meditaed migration, invasion and metastasis of gastric cancer

**Figure 4:**
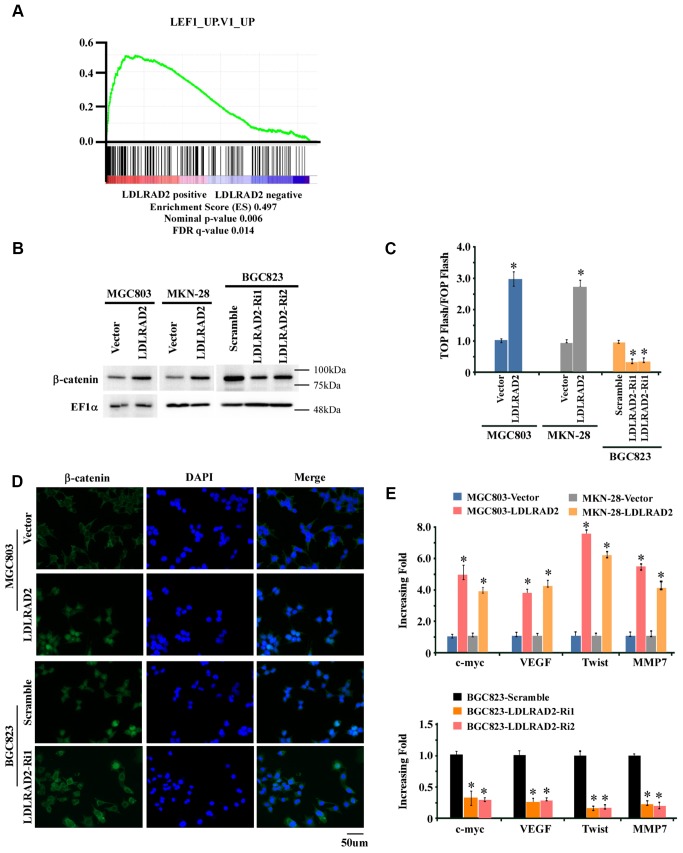
**LDLRAD2 activates Wnt/β-catenin signaling pathway in GC.** Gene-set enrichment analysis (GSEA) indicated that LDLRAD2 expression was closely associated with Wnt/β-catenin pathway (**A**). Western blotting analysis showed that overexpression of LDLRAD2 significantly increased the nuclear expression of β-catenin, while silence of LDLRAD2 dramatically decreased the nuclear expression of β-catenin in GC cell lines (**B**). Top/Fop flash assay also demonstrated that overexpression of LDLRAD2 significantly activated Wnt/β-catenin signaling pathway, while silence of LDLRAD2 dramatically restrained Wnt/β-catenin signaling pathway (**C**). Immunofluorescence assay also showed that LDLRAD2 could promote the nuclear distribution of β-catenin in GC cells (**D**). Overexpression of LDLRAD2 significantly promoted the expression of downstream genes of Wnt/β-catenin signaling including c-myc, VEGF, Twist, and MMP7, while silence of LDLRAD2 inhibited the expression of these genes (**E**).

To explore the potential molecular mechanism underlying the accelerative effect of LDLRAD2 on the migration, invasion and metastasis of GC, firstly we performed gene-set enrichment analysis (GSEA) to screen the LDLRAD2-associated signaling pathway. As shown in Figure 4A, GSEA suggested that there may be a close relationship between LDLRAD2 and Wnt/β-catenin signaling pathway. Consistently, our western blotting analysis showed that overexpression of LDLRAD2 significantly increased the nuclear expression of β-catenin, while silence of LDLRAD2 dramatically decreased the nuclear expression of β-catenin in GC cell lines (Figure 4B). Similarly, our Top/Fop flash assay also demonstrated that overexpression of LDLRAD2 significantly activated Wnt/β-catenin signaling pathway, while silence of LDLRAD2 dramatically restrained Wnt/β-catenin signaling pathway (Figure 4C). Additionally, using immunofluorescence assay we further confirmed that LDLRAD2 could promote the nuclear distribution of β-catenin in GC cells (Figure 4D). Moreover, we observed that overexpression of LDLRAD2 substantially upregulated the expression of downstream genes of Wnt/β-catenin such as c-myc, VEGF, Twist, and MMP7, while silence of LDLRAD2 inhibited the expressions of these genes (Figure 4E). Taken together, these results demonstrated that LDLRAD2 is involved in the activation of Wnt/β-catenin signaling pathway in GC cells.

**Figure 5:**
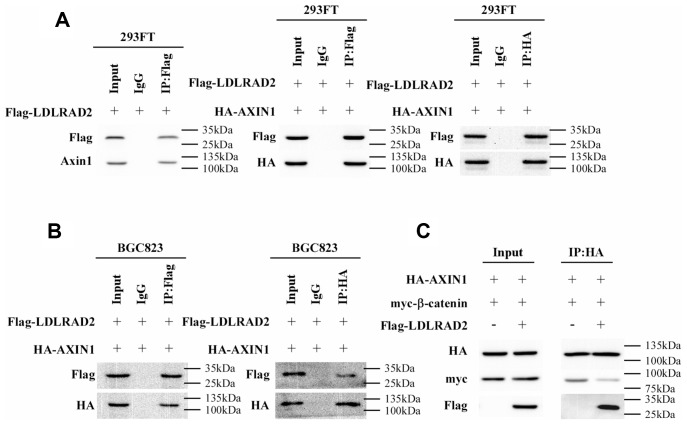
**LDLRAD2 activates Wnt/β-catenin signaling pathway by targeting Axin1 in GC.** Co-immunoprecipitation assay showed that LDLRAD2 could bind to Axin1 (**A**). LDLRAD2 inhibited Axin1 from binding to β-catenin (**B**–**C**).

Then we further tried to elucidate the mechanism responsible for the LDLRAD2-induced activation of Wnt/β-catenin signaling pathway in GC cells. Nuclear translocation of β-catenin, is a typical hallmark of activated Wnt/β-catenin signaling pathway. Degradation of cytoplasmic β-catenin is a pivotal mechanism of inhibiting the nuclear translocation of cytoplasmic β-catenin, which negatively regulates Wnt/β-catenin signaling pathway. The destruction complex (DC) consisting of the tumor suppressor protein Axin1, APC, β-catenin and two constitutively active serine-threonine kinases (CK1α/δ and GSK3α/β) has been demonstrated to participate in the degradation of cytoplasmic β-catenin. Of these components, Axin1 plays a crucial role in maintaining the DC activity of degrading cytoplasmic β-catenin. Therefore, we hypothesized that LDLRAD2 might activate Wnt/β-catenin signaling pathway by targeting Axin1. Strikingly, co-immunoprecipitation assay showed that LDLRAD2 could bind to Axin1 (Figure 5A). Furthermore, the interaction between LDLRAD2 and Axin1 significantly prevented Axin1 from binding to β-catenin (Figure 5B–5C), which largely reduced the degradation of cytoplasmic β-catenin. Overall, these findings suggested that LDLRAD2 interacted with Axin1 to inhibit it from binding to cytoplasmic β-catenin, which inhibited the degradation of β-catenin and thereby facilitated its nuclear translocation, ultimately activating Wnt/β-catenin signaling pathway.

**Figure 6:**
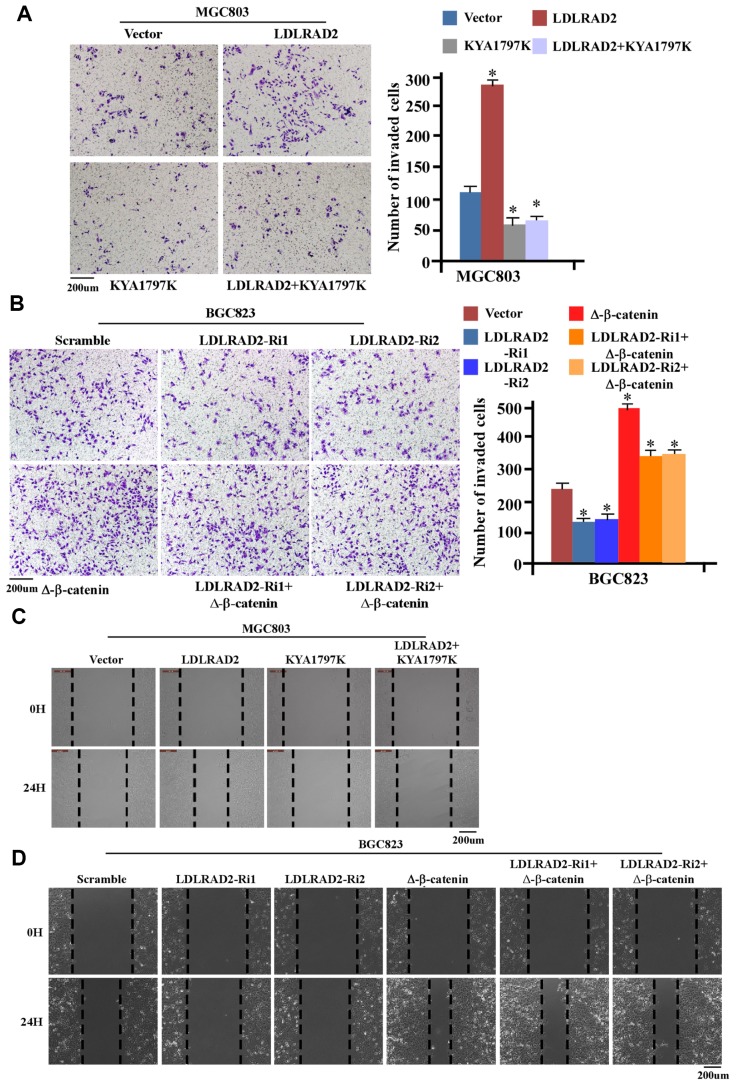
**LDLRAD2-dependent activation of Wnt/β-catenin signaling promotes migration and invasion of GC cells**. KYA1797 significantly inhibited the LDLRAD2-induced invasion of GC cells (**A**), while Δ-β-catenin markedly reversed the inhibitory effect of silence of LDLRAD2 on the invasion of GC cells (**B**). KYA1797 dramatically mitigated LDLRAD2-induced migration of GC cells (**C**), while Δ-β-catenin obviously reversed the inhibitory effect of silence of LDLRAD2 on the migration of GC cells (**D**).

We further explored whether Wnt/β-catenin signaling pathway was involved in the effect of LDLRAD2 on migration, invasion and metastasis of GC cells. We observed that KYA1797, a Wnt/β-catenin signaling inhibitor, could significantly mitigate the accelerative effect of LDLRAD2 overexpression on the invasion of MGC803 cells (Figure 6A), while Δ-β-catenin, the activated form of β-catenin, could substantially inhibit the inhibitive effect of LDLRAD2 silence on the invasion of BGC823 cells (Figure 6B). Also, our wound healing assay showed that KYA1797 could dramatically counteract the accelerative effect of LDLRAD2 overexpression (Figure 6C), while Δ-β-catenin markedly decreased the inhibitive effect of LDLRAD2 silence on the migration of GC cells (Figure 6D). The similar results of migration and invasion were observed when KYA1797 was used to treat MKN-28 cells (Supplementary Figure 2). Taken together, these results demonstrated that LDLRAD2 promoted the migration and invasion of GC cells by activating Wnt/β-catenin signaling pathway.

## DISCUSSION

The tumorigenesis of GC is a complex process that involves aberrant expressions of multiple genes [25]. In recent years, an increasing number of studies focused on identifying novel tumorigenesis-associated genes by sequencing technique such as transcriptome sequencing and proteomic sequencing [26–28]. In this study, we found that LDLRAD2 expression was significantly upregulated in GC tissues compared with adjacent normal tissues, and it was closely correlated with positive lymph node metastasis, distant metastasis and poor survival in GC patients. Using in vitro and in vivo models, we further observed that LDLRAD2 overexpression promoted the epithelial-mesenchymal transition (EMT), migration, invasion and metastasis of GC cells. Additionally, it was found that LDLRAD2 could interact with Axin1, which facilitated the nuclear translocation of β-catenin and then activated Wnt/β-catenin signaling pathway. Hence, these results suggested that LDLRAD2 may act as a pro-oncogene in GC development.

The EMT, a highly conserved cellular program, can induce epithelial cells to lose their cell polarity and cell-cell adhesion, by which epithelial cells transform into mesenchymal cells with migratory and invasive properties [15, 16]. EMT participates in maintaining many physiologic processes such as mesoderm formation and neural tube formation [29], and is also involved in numerous disease processes including organ fibrosis, wound healing as well as initiation of tumor invasion and metastasis [30]. In our study, GSEA-GO analysis of TCGA data suggested that LDLRAD2 expression was closely associated with EMT of GC cells. Furthermore, our functional experiments in vitro showed that overexpression of LDLRAD2 induced GC cells to grow in a spindle shape. In addition, we found that silence of LDLRAD2 led to upregulation of epithelial markers including γ-catenin, Snail, Slug and E-cadherin, but downregulation of mesenchymal marker N-cadherin and vimentin, overexpression of LDLRAD2 obtained the opposite results. These results revealed that LDLRAD2 could induce EMT of GC cells. Solid evidence shows that EMT exhibits accelerative effects on dissemination of human malignancies, thereby contributing to cancer progression [31, 32]. In accordance with this, our functional experiments showed that overexpression of LDLRAD2 significantly facilitated migration, invasion and metastasis of GC cells, whereas downregulation of LDLRAD2 inhibited these processes. Taken together, these results suggested that LDLRAD2 facilitated migration, invasion and metastasis of GC cells probably by inducing EMT.

Numerous cancer-associated signaling pathways are involved in tumor invasion and metastasis [33–35]. To investigate the underlying mechanisms for the LDLRAD2-dependent migration, invasion and metastasis of GC cells, firstly we performed gene-set enrichment analysis (GSEA) based on TCGA data to screen the signaling pathway associated with LDLRAD2. The results of GSEA suggested that LDLRAD2 might regulate Wnt/β-catenin signaling pathway in GC. It has been proven that aberrant activation of Wnt/β-catenin pathway can promote EMT, invasion and metastasis of various cancers including GC [17, 36]. Therefore, we herein asked if LDLRAD2 contributed to GC invasion and metastasis by activating Wnt/β-catenin signaling pathway. Increased nuclear expression of β-catenin is the hallmark of the activation of Wnt/β-catenin signaling pathway, which is largely dependent on the nuclear translocation of cytoplasmic β-catenin [17, 36]. Our results showed that overexpression of LDLRAD2 significantly increased the nuclear expression of β-catenin, while silence of LDLRAD2 dramatically decreased the nuclear expression of β-catenin of GC cells, suggesting LDLRAD2 acted as an activator of Wnt/β-catenin pathway. Additionally, we observed that overexpression of LDLRAD2 resulted in significant upregulation of the downstream genes of Wnt/β-catenin such as c-myc, VEGF, Twist, and MMP7, while silence of LDLRAD2 showed the opposite effects, which further demonstrated that LDLRAD2 could activate Wnt/β-catenin pathway in GC cells. More importantly, it was found that Wnt/β-catenin signaling inhibitor could reverse the accelerative effect of LDLRAD2 overexpression on the in vitro migration and invasion of GC cells, while Δ-β-catenin could reverse the inhibitory effect of LDLRAD2 downregulation on the in vitro migration and invasion of GC cells. Overall, these results suggested that LDLRAD2 facilitated GC invasion and metastasis via activation of Wnt/β-catenin pathway. Of course, in vivo functional experiments should be performed to further validate the key role of Wnt/β-catenin pathway in LDLRAD2-induced invasion and metastasis in future. Evidence showed that activation of Wnt/β-catenin pathway promoted cell invasion and metastasis by inducing EMT in various cancers including GC [20–24, 37]. Albeit in this study we did not explore the effect of inhibition of EMT on LDLRAD2/Wnt/β-catenin signaling axis-induced invasion and metastasis of GC cells, we may deduce that LDLRAD2 activates Wnt/β-catenin to induce EMT, thereby promoting invasion and metastasis of GC cells. Of note, abnormal activation of Wnt/β-catenin pathway has been demonstrated to facilitate cancer progression by regulating multiple other processes of tumor phenotypes such as proliferation, stem cell-like properties, chemotherapy resistance and angiogenesis, in addition to invasion and metastasis [38–45]. Therefore, in future studies it is very interesting and meaningful to further explore whether LDLRAD2 can also promote proliferation, drug resistance, angiogenesis and stem cell-like properties, or inhibit apoptosis of GC cells, because this would help us to get a thorough understanding of the role of LDLRAD2-induced GC progression.

Nuclear translocation of cytoplasmic β-catenin that increases the expression of nuclear β-catenin is a prerequisite event for the activation of Wnt/β-catenin pathway [17, 36]. Hence, we hypothesized that LDLRAD2 may activate Wnt/β-catenin pathway by facilitating nuclear translocation of cytoplasmic β-catenin. The degradation of cytoplasmic β-catenin has been proven to impede the nuclear translocation of cytoplasmic β-catenin, thereby negatively meditating canonical Wnt/β-catenin pathway [17, 36]. The destruction complex (DC) consisting of Axin1 and APC, β-catenin and two constitutively active serine-threonine kinases (CK1α/δ and GSK3α/β) plays a crucial role in degrading cytoplasmic β-catenin [38–45]. Axin1 is a key component favoring the DC activity and many upstream molecules of Wnt/β-catenin pathway could meditate its activation by targeting Axin1 in tumor cells [46–48]. Similar to these previous studies, in current study we also found that LDLRAD2 could bind to and inhibit Axin1 from binding to cytoplasmic β-catenin, which largely mitigate the degradation of cytoplasmic β-catenin thereby activating Wnt/β-catenin pathway.

In summary, our findings demonstrate that high LDLRAD2 expression strongly correlates tumor aggressiveness and poor prognosis, and LDLRAD2 activates Wnt/β-catenin pathway by interacting with Axin1 to EMT, invasion and metastasis of GC cells. In conclusion, these findings indicate that LDLRAD2/Wnt/β-catenin axis may be a potential therapeutic target for GC patients.

## MATERIALS AND METHODS

### Clinical tissue samples

A total of 180 GC patients, who received radical gastrectomy and D2 lymphadenectomy followed with postoperative chemotherapy from April 2010 to June 2012, were histopathologically and clinically diagnosed at Lanzhou University Second Hospital. All tumor tissues were confirmed as GC using hematoxylin and eosin (H&E) staining after surgical resection. The clinicopathologic staging of the patients were determined according to the American Joint Committee on cancer criteria. Normal GC tissues were obtained from a standard distance (2 cm) from the margin of resected neoplastic tissues of patients with GC cancer and confirmed by pathological evaluation. All the patients did not receive any anti-cancer therapy, such as chemotherapy and radiotherapy before surgical treatment. All patients were followed up for at least 5 years. This study was approved by the Institute Research Ethics Committee of Lanzhou University Second Hospital and the approved study protocol number is 2019A-100. Written informed consent was signed by each patient. We performed all experiments using these samples strictly based on relevant regulations and laws. The clinical parameters of GC patients are summarized in Table 1.

### Bioinformatics analysis

The Cancer Genome Atlas (TCGA) database is an online tool Gene Expression Profiling Interactive Analysis (GEPIA) (http://gepia.cancer-pku.cn/). In this study, we analyzed TCGA database to compare the mRNA levels of LDLRAD2 between in 29 GC tissues and in 29 normal tissues (TCGA-STAD). Additionally, OS and DFS analyses of GC patients in the TCGA-STAD database were performed using the online tool GEPIA and the results were downloaded from the website. To explore the association of LDLRAD2 with cancer-associated signaling pathways, Global mRNA expression profiles of a subset of TCGA GC specimens were subjected to Gene set enrichment analysis (GSEA). The GSEA was fulfilled using GSEA software version 3.0. GSEA-GO analysis was performed using GSEA against c6.oncogenic signatures. gmt. The normalized enrichment score is the primary statistic for examining gene set enrichment results.

### Cell culture

Human gastric cell lines, including GES-1, AGS, MKN-28, BGC823, MKN45, MGC45, MGC803, SGC7901 and HGC-27 were obtained from the Chinese Academy of Science Committee Type Culture Collection Cell Bank (Shanghai, China). All cell lines were cultured in Roswell Park Memorial Institute 1640 (RPMI-1640) medium with supplementation of 10% fetal bovine serum(FBS) (Invitrogen, Carlsbad, CA, USA) and appropriate amounts of penicillin (100U/ml) and streptomycin (100mg/ml). Cells were incubated in a humidified atmosphere of 5% CO_2_ at 37°C.

### Plasmid construction and RNA interference

The LDLRAD2 ORF sequence was amplified and cloned into LV003-IRES-EGFP (Forevergen Biosciences Co., Ltd., Guangzhou, China). Lentiviruses were produced by co-transfecting the constructed plasmid with the packaging plasmids psPAX2 and pMD2.G (Addgene, Watertown, MA) into 293T cells using Lipofectamine 2000 (Invitrogen, Carlsbad, CA) for approximately 72 hours. Next, we collected culture supernatants for filtration and concentration, and then we used the concentrate to infect MGC-803 and MKN28 cells. After 48 hours, the infected cells were selected by 2 μg/mL puromycin (540411; Merck Millipore, Burlington, MA), and successful establishment was validated using western blotting analysis.

Two small interfering RNA (siRNA) duplexes targeting LDLRAD2 were bought from RiboB (Guangzhou, China). Then, we designed two shRNAs targeting LDLRAD2 based on the above siRNAs. BGC-823 cells were chosen to establish LDLRAD2-silenced GC cells.

### Cell invasion and wound healing assays

Transwell Matrigel invasion assay was performed as previously described. After incubation for 24 hours, cells invading to the lower chambers were fixed, stained, photographed and quantified by counting them in five random 200× magnification fields. As to wound healing assay, we first starved cells seeded in six-well plates for 12 hours to leave them in cell cycle synchronization, and then scratched the confluent monolayer of cells with sterile 200-ml pipette tips for artificially creating wounds. The wound healing process was observed and photographed under 100× magnification at indicated time points.

### Animal studies

To explore the role of LDLRAD2 in regulating GC metastasis, we evenly divided 25 BALB/c nude mice into 5 groups in a random manner. 2 × 10^6^ cells with MGC803-Vector, MGC803-LDLRAD2, BGC823-Scramble, BGC823-LDLRAD2-Ri1 or BGC823-LDLRAD2-Ri2 were injected intravenously through tail vein into nude mice. All mice were sacrificed for excision of lungs 6 weeks after injection. The surface metastatic sites on lung surface were counted. Animal protocols were approved by the Institutional Animal Care and Use Committee of Lanzhou University Second Hospital. Moreover, all animal experiments were also performed based on the Declaration of Helsinki and the guidelines established by the National Institutes of Health Guide for the Care and Use of Laboratory Animals.

### TOP/FOP flash assay

Cells were seeded in triplicate in 24-well plates. After 24 hours, indicated plasmids (TOP flash or FOP flash) plus 1 ng pRL-TK Renilla plasmid were transfected into the cells using Lipofectamine 3000 Reagent (Life Technologies). 48 hours after transfection, Dual-Luciferase Reporter Assay (Promega) was performed according to the manufacturer’s instructions, as previously described [49].

### Western blot and immunofluorescence assays

Protein was electrophoretically separated by 10% SDS-PAGE and transferred to PVDF membranes (Millipore, Billerica, MA, USA). The membranes were blocked for 2 hours with 5% skim milk in TBST at room temperature, and then incubated with specific primary antibodies (anti-LDLRAD2, anti-γ-catenin, anti-E-cadherin, anti-Vimentin, anti-N-cadherin, and anti-GAPDH) overnight at 4 °C. After incubated with primary antibodies, the membranes were washed with TBST for three times, incubated with rabbit or mouse radish peroxidase-coupled secondary antibodies for 2 hours at room temperature, and then washed with TBST for three times. Antibody binding was detected by the enhanced chemiluminescence (Pierce, Waltham, MA). Immunofluorescence staining assay was performed using anti-β-catenin (dilution 1:200) and FITC-conjugated goat anti-Rabbit secondary antibody (Thermo Fisher, Waltham, MA). Images were recorded with a fluorescence microscope.

### Total RNA extraction and RT-PCR analysis

Samples were lysed with Trizol reagent (Invitrogen, Life Technologies), then total RNA was isolated based on the manufacturer’s instructions. The extracted RNA was re-suspended in RNase-free water, and 1 μg RNA from each sample was used for cDNA synthesis primed with random hexamers. Then we used the synthesized cDNAs for Quantitative RT-PCR. All Quantitative RT-PCR reactions were fulfilled using LightCycler 480 (Roche). The fold change in expression was calculated using the 2-ΔΔCt method, in which with the GAPDH mRNA was used as an internal control. PCR amplification was performed using the SYBR Green PCR master mix Kit (Promega, USA). The primer sequences were as follows: GAPDH forward, 5′-GACTCATGACCACAGTCCATGC-3′, GAPDH reverse, 5′ AGAGGCAGGGATGATGTTCTG-3′ MYC forward, 5′- GGCTCCTGGCAAAAGGTCA-3′, MYC reverse, 5′-CTGCGTAGTTGTGCTGATGT-3′ TWIST forward, 5′-TCCATTTTCTCCTTCTCTGGAA-3′, TWIST reverse, 5′-GTCCGCGTCCCACTAGC-3′ MMP7 forward, 5′-GAGTGAGCTACAGTGGGAACA-3′, MMP7 reverse, 5′ CTATGACGCGGGAGTTTAACA T-3′ VEGF forward, 5′-CTACCTCCACCATGCC AAGT-3′, VEGF reverse, 5′-AGCTGCGCTGATAGA CATCC-3′.

### Co-immunoprecipitation analysis

Cells were transfected with various constructs and washed with PBS after 48hours, then lysed in an NP-40-containing lysis buffer supplemented with protease inhibitor cocktail (Roche), and then immunoprecipitated, as instructed by the protocol of a standard method, as previously described [50], with the indicated antibodies.

### Immunohistochemical (IHC) staining

Immunohistochemistry assays were performed on the paraffin-embedded GC samples, using the following primary antibodies: anti-LDLRAD2 (1:150; Abcam) and anti-b-catenin (1:200; Abcam). Three pathologists assessed the staining intensity and percent of each section independently, and graded it according to the criteria as described below. The staining intensity score was evaluated as: 0 = negative, 1 = weak, 2 = moderate, and 3 = strong. The staining area score was evaluated as: 0 = 0%, 1 = 1-25%, 2 = 26-50%, 3 = 51-75%, and 4 = 76-100%. The scores of staining intensity and staining area were multiplied to produce the final score. The final staining scores ≤ 3 were regarded as low LDLRAD2 or β-catenin expression and the scores ≥ 4 were regarded as high expression.

### Statistical analysis

Data analysis was performed by using the SPSS 19.0 software (SPSS Inc., Chicago, IL, USA). Comparisons between any two groups were conducted using Student’s t-test (two-sided). To assess the association of LDLRAD2 expression with clinic-pathologic parameters of GC patients, they were divided into high and low LDLRAD2 expression groups according to the median value of LDLRAD2 expression. The clinic-pathologic parameters of GC patients between high and low LDLRAD2 expression was compared using chi-squared test. Kaplan-Meier method was used to establish survival curves for OS and DFS. The differences in survival of patients between high and low LDLRAD2 expression were assessed by log-rank test. P < 0.05 was regarded as statistically significance.

## Supplementary Material

Supplementary Figures
