# Supplementary material for: LDLRAD2 overexpression predicts poor prognosis and promotes metastasis by activating Wnt/β-catenin/EMT signaling cascade in gastric cancer
Source: Aging (Albany NY). 2019 Oct 24;11(20):8951–68. doi: 10.18632/aging.102359 (PMC6834412; doi:10.18632/aging.102359)
Supplement: Supplementary Figures [file aging-11-102359-s001.pdf]

SUPPLEMENTARY FIGURES

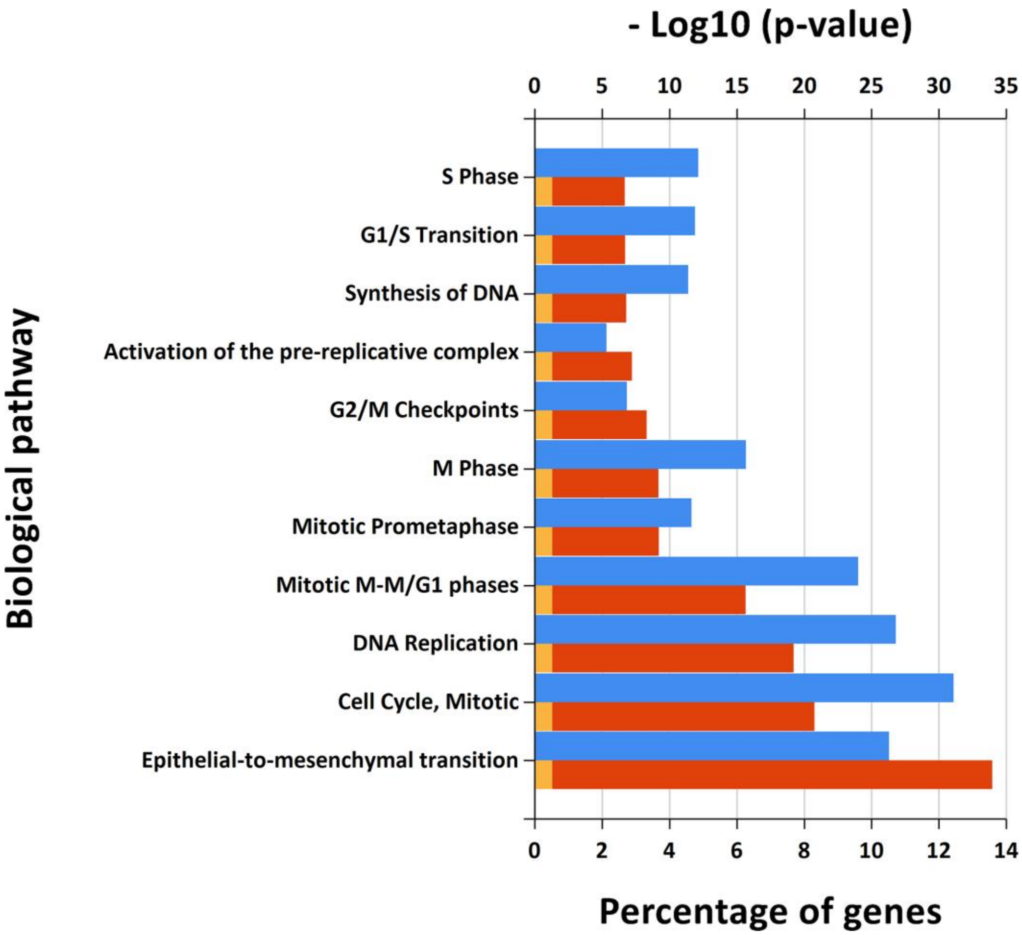

Supplementary Figure 1. Gene-set enrichment analysis (GSEA)-GO analysis based on TCGA data suggests that LDLRAD2 correlates with EMT.

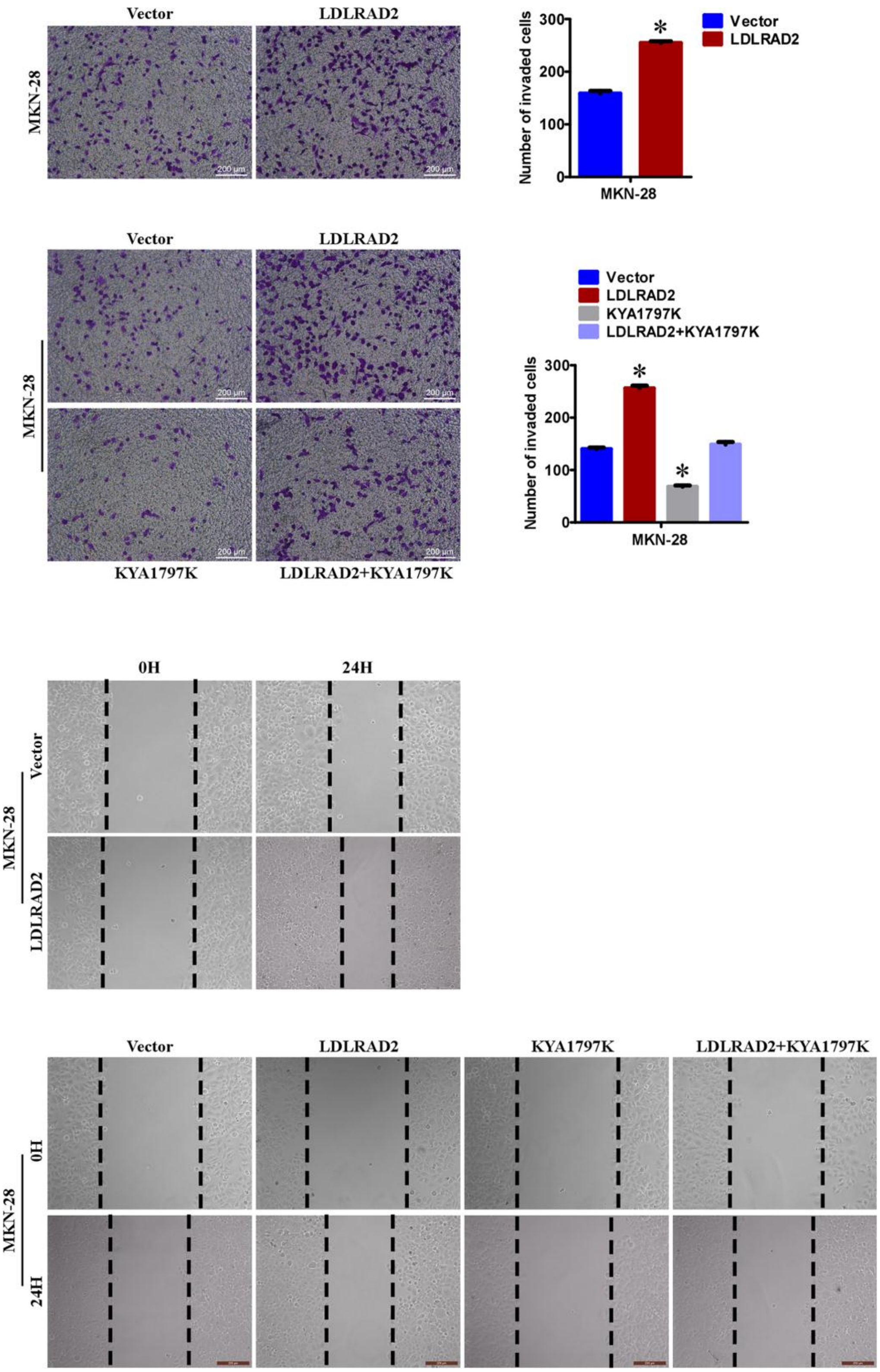

Supplementary Figure 2. LDLRAD2 promotes the migration and invasion of MKN-28 cells by activating Wnt/β-catenin signaling pathway.
